# Case Report: High-volume, aggressive prostate cancer with borderline psa levels diagnosed by PSMA PET/CT–based comprehensive radiographic scheme and cognitive fusion targeted biopsy

**DOI:** 10.3389/fonc.2025.1721477

**Published:** 2025-12-01

**Authors:** Yiheng Zhou, Renyuan Qiu, Mengmeng Dou, Wenkai Bi, Yong Yu

**Affiliations:** 1 Department of Urinary Surgery, The 960th Hospital of the PLA Joint Logistics Support Force, Jinan, Shandong, China; 2 Department of Radiology, Shandong Rongjun General Hospital, Jinan, Shandong, China; 3 Department of Neurology, Tianjin First Central Hospital, Tianjin, China; 4 Department of Nuclear Medicine, Shandong Provincial Hospital affiliated to Shandong First Medical University, Jinan, Shandong, China; 5 Department of Ultrasound, Shandong Provincial Hospital affiliated to Shandong First Medical University, Jinan, Shandong, China

**Keywords:** prostate cancer, radiographic diagnosis, prostate-specific antigen, PSMA PET/CT, therapeutic outcomes

## Abstract

Prostate-specific antigen (PSA) is a widely used biomarker for prostate cancer (PCa), but its limitations are evident in patients with PSA at boundary level. We present a 61-year-old male with gross hematuria and a PSA level of 7.25 ng/mL with an fPSA/tPSA ratio of 0.19, initially suspected of a bladder tumor. Conventional imaging, including ultrasound, CT, and MRI, revealed suspicious prostate and pelvic lesions but failed to delineate the degree of disease involvement. Prostate-specific membrane antigen positron emission tomography/computed tomography (PSMA PET/CT) demonstrated extensive multi-organ involvement, including in the pelvic lymph nodes, bone, lung, and pleura, enabling accurate staging. On the basis of PSMA PET/CT examination, we conducted targeted cognitive fusion biopsy. Biopsy confirmed high-grade acinar adenocarcinoma with Gleason scores of predominantly Gleason score 9 (4 + 5) disease. The patient received chemotherapy with docetaxel and nedaplatin followed by combined endocrine therapy, achieving rapid PSA decline and radiographic disease stabilization. This case demonstrates the insufficiency of PSA as a sole diagnostic marker and highlights the pivotal role of PSMA PET/CT in detecting occult metastases, assisting in targeted and precise biopsy, guiding staging, and optimizing individualized therapy. Furthermore, these findings emphasize the necessity of a comprehensive radiographic scheme in which conventional imaging provides initial screening and PSMA PET/CT confirms the extent of disease, ensuring precise diagnosis and tailored treatment for borderline PSA but aggressive PCa.

## 1 Introduction

Prostate cancer (PCa) is one of the most prevalent malignancies in men and is commonly diagnosed through methods such as digital rectal examination (DRE), elevated prostate-specific antigen (PSA) levels, and radiographic examinations, with definitive diagnosis relying on biopsy (1–5). PSA levels have traditionally played a pivotal role in the diagnostic process of PCa; however, their limitations have become increasingly evident in current medicine, as PCas exhibiting low or borderline PSA levels may fail to be detected (6, 7). Typically, PSA levels above 10 ng/mL or values between 4 and 10 ng/mL with a low free-to-total PSA ratio (fPSA/tPSA) suggest the need for further investigation (8). Nevertheless, such thresholds may fail to identify patients whose PSA remains within the normal or mildly elevated range, in which several patients may even exhibit aggressive or metastatic forms of the disease. Generally speaking, the degree of invasion of prostate cancer is positively correlated with the elevated level of PSA. However, the elevated level of PSA in some patients with aggressive prostate cancer is not consistent with their tumor load. For these patients, simply referring to PSA level may underestimate the clinical tumor progression of patients. Furthermore, many patients demonstrate high-grade pathological features but actually produce less PSA than low-grade PCas (9, 10). These clinical challenges underscore the imperative for advanced diagnostic tools, particularly in cases where traditional biomarkers such as PSA exhibit limited reliability, thereby necessitating the integration of effective radiographic technologies to improve diagnostic accuracy (11).

Prostate-specific membrane antigen positron emission tomography-computed tomography (PSMA PET/CT) has emerged as a transformative modality for imaging PCa, particularly for identifying ambiguous metastatic lesions (12). Its high sensitivity for PSMA expression enables superior detection of metastatic disease even in patients with low or borderline PSA levels (13). This technique offers significant advantages in accurately staging high-risk cases, guiding diagnosis and therapy, and assessing treatment response. In addition, PSMA PET/CT can assist in targeted puncture of prostate lesions, which can significantly improve the detection rate of prostate tumors and reduce complications such as hematuria and infection compared with traditional systematic core needle biopsy (14). thus, it may benefit patients with high-volume, aggressive prostate cancer with a disproportionately low PSA for its stage, where conventional diagnostic approaches provide insufficient medical evidence.

In this study, we present a unique case of advanced multi-metastatic prostate cancer diagnosed through comprehensive radiographic examinations, despite a PSA rising to the borderline range. This patient exhibited multiple metastatic lesions, including bladder invasion and multifocal pulmonary and skeletal involvement, which would typically be considered atypical for a PCa patient with a PSA rising to the borderline range. Initial diagnostic imaging technologies, including ultrasound, computed tomography (CT) and magnetic resonance imaging (MRI), provided several indications of abnormalities; however, PSMA PET/CT was instrumental in confirming the presence of primary prostate cancer and extensive metastases and assisting in precise staging and following appropriate therapeutic regimens for the disease. Subsequently, PSMA PET/CT targeted cognitive fusion puncture biopsy technology obtained prostate living tissue accurately and minimally invasive, which benefited patients clinically.

## 2 Case presentation

**Figure 1:**
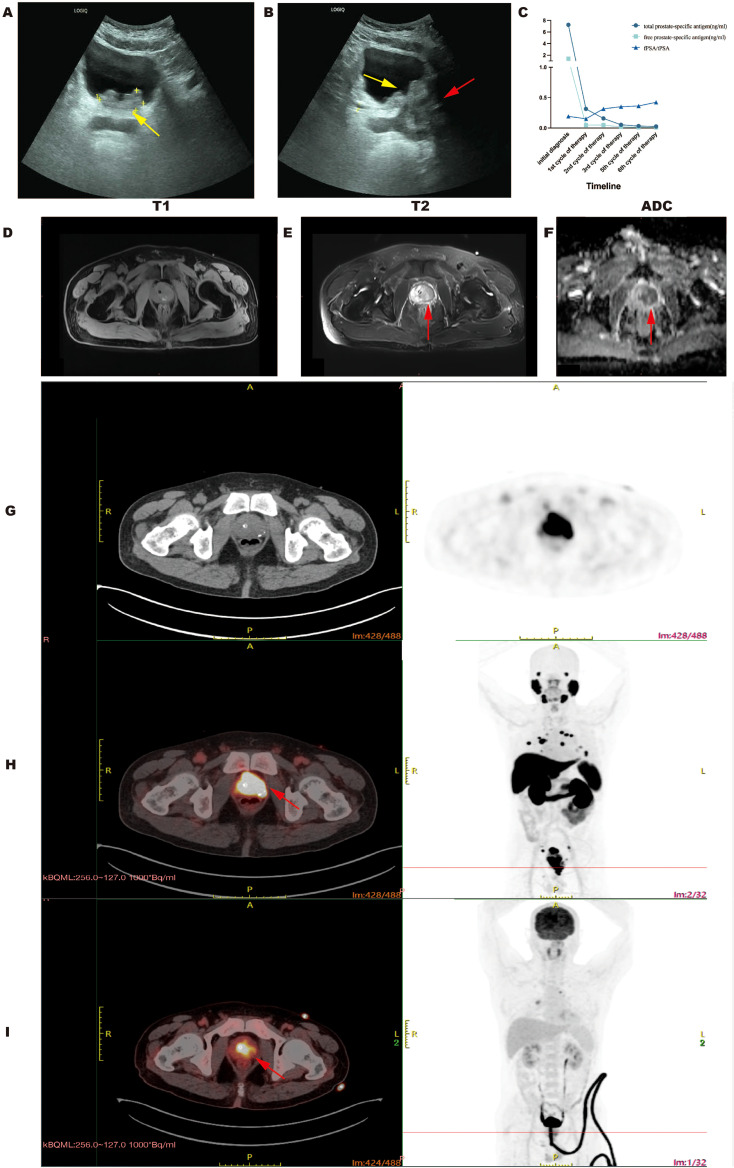
Serum PSA levels and typical radiographic characteristics of the primary PCa site. **(A, B)** Ultrasonographic images demonstrating intravesical involvement (yellow arrow) and the prostatic lesion (red arrow). **(C)** Line chart illustrating the dynamic changes in total prostate-specific antigen (tPSA, reference range 0–5 ng/mL), free prostate-specific antigen (fPSA, reference range 0–1 ng/mL), and the fPSA/tPSA ratio (reference range 16%–100%) during the diagnostic and therapeutic period. **(D–F)** The MRI features of the prostatic lesion (red arrow) are hypointense on the T1-weighted image **(D)**, hyperintense on the T2-weighted image **(E)**, and restricted on the apparent diffusion coefficient (ADC) map **(F)**. **(G–I)** Representative PET/CT and CT images of the prostate: **(G)** CT scan, **(H)** PSMA PET/CT and **(I)** FDG PET/CT. Red arrows show markedly elevated PSMA uptake (SUVmax 41.3) **(H)** and mildly increased FDG uptake (SUVmax 6.9) **(I)** at the PCa site.

**Figure 2:**
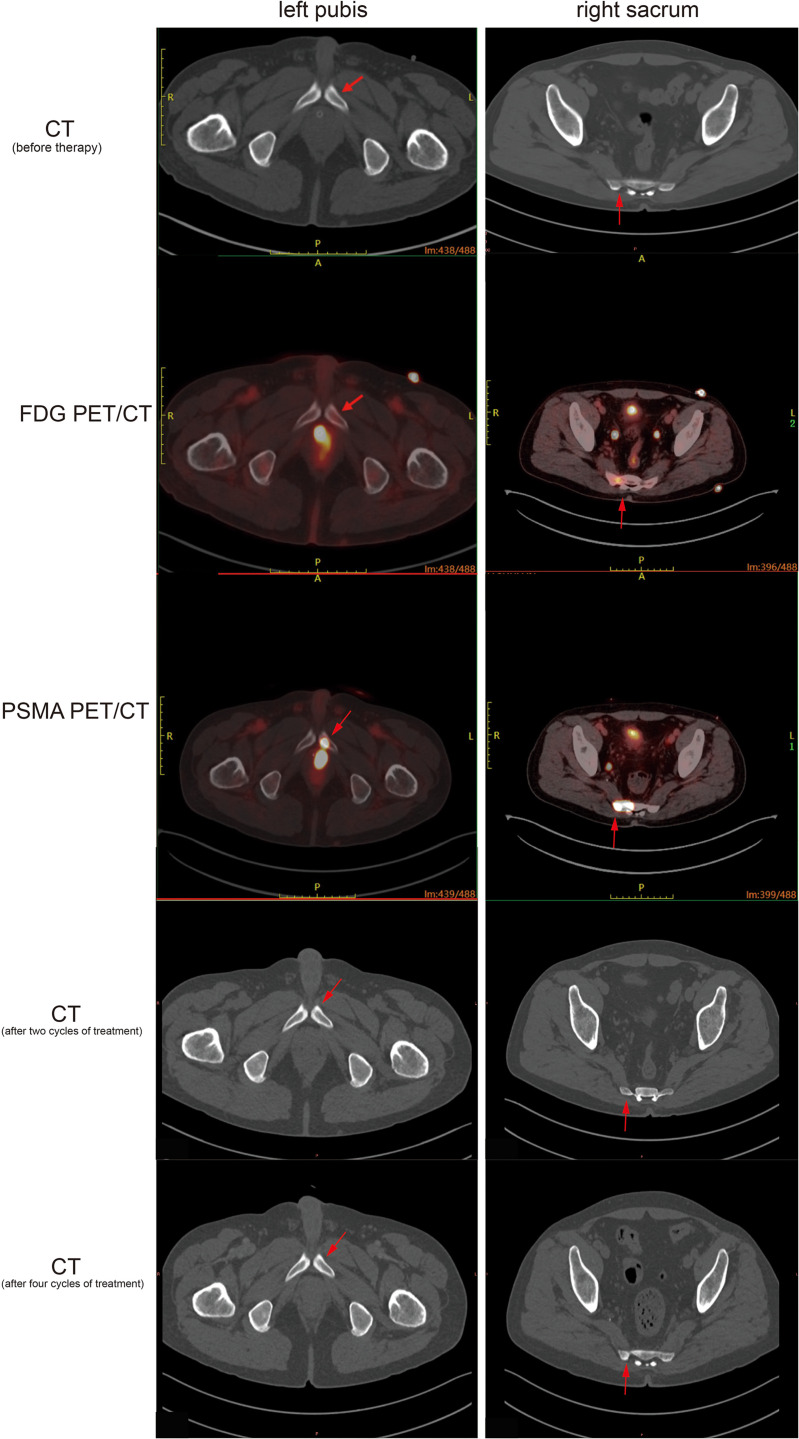
Representative PET/CT and CT images demonstrating PCa metastases in the left pubis and right sacrum with markedly elevated PSMA uptake (SUVmax 24.3 and 41.3, respectively) and only mildly increased FDG uptake (SUVmax 2.0 and 4.7, respectively), while no obviously identifiable metastatic lesions were shown on the corresponding CT images, which remained stable throughout the whole treatment period.

**Figure 3:**
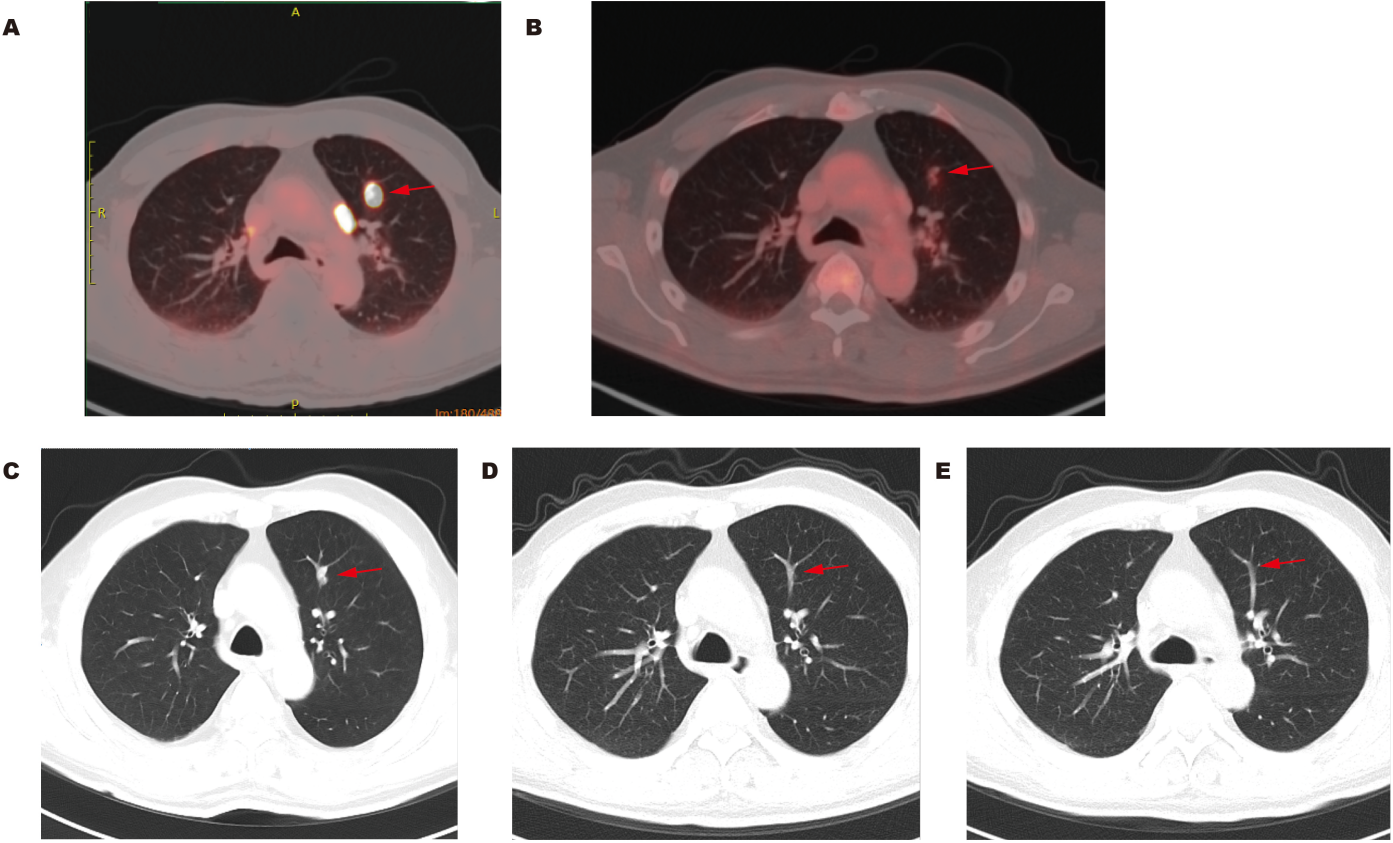
Representative PET/CT and CT images of lungs before and after treatment. **(A)** Lung metastasis (red arrow) showing markedly elevated PSMA uptake (SUVmax 21.2) before treatment; **(B)** Lung metastasis (red arrow) showing only mildly increased FDG uptake (SUVmax 2.2) before treatment; **(C)** CT image of the same lung metastatic site (red arrow) at the corresponding plane with PET/CT before treatment; **(D)** CT image showing a significant reduction in the same lung metastatic site (red arrow) at the corresponding plane after two cycles of treatment; **(E)** CT image showing a significant reduction in the same lung metastatic site (red arrow) at the corresponding plane after four cycles of treatment.

A 61-year-old male patient with gross hematuria presented at our hospital in October 2023. Ultrasound examination revealed a cauliflower-like slightly hyperechoic lesion on the posterior wall of the bladder that protruded into the bladder cavity. The patient was admitted with an initial diagnosis of “bladder tumor” (**Figures 1A, B**). Upon hospitalization, comprehensive auxiliary examinations were conducted. The blood PSA levels indicated: total prostate-specific antigen (tPSA) 7.25 ng/mL, free prostate-specific antigen (fPSA) 1.4 ng/mL, and an fPSA/tPSA of 0.19 (**Figure 1C**). The patient also underwent other imaging examinations because of the borderline range rising PSA. Prostate MRI revealed prostatic hyperplasia with an abnormal signal lesion in the left lobe, with strong suspicion of prostate cancer (**Figures 1D–F**). Because the primary origin of the malignancy was uncertain, a whole-body FDG PET/CT was first performed to evaluate the potential presence of metastases, assess possible systemic disease and exclude other potential malignancies or inflammatory conditions. The scan revealed increased FDG uptake in the prostate and left pubic bone, along with mildly elevated metabolism in multiple pulmonary nodules and pleural thickenings (**Figures 1G–I**). Additionally, widespread variable FDG activity was observed throughout the lymph nodes in the pelvis, mediastinum, and hilum, primarily suggesting possible systemic metastases. To achieve higher diagnostic precision, a PET 18F-PSMA-1007 scan was further conducted, revealing a focally increased uptake of the prostate with an irregular shape and indistinct borders from the bladder and seminal vesicles (**Figures 1G–I**). High PSMA uptake was also observed at other sites, which revealed the following findings:

1. Multiple lymph nodes in the pelvic area presented high PSMA uptake and slightly elevated FDG metabolism (**Figure 2**).
2. The sacrum exhibited no significant metastatic lesions on CT but demonstrated heterogeneous high PSMA uptake (SUVmax 41.3) with only mildly increased FDG metabolism (SUVmax 4.7) (**Figure 2**).
3. The left pubis also showed no obvious metastatic lesions on CT but displayed high PSMA uptake (SUVmax 24.3) with slightly increased FDG metabolism (SUVmax 2.0) (**Figure 2**).
4. Multiple nodules in both lungs exhibited high PSMA uptake and slightly elevated FDG metabolism (**Figures 3A, B**).
5. Multiple lymph nodes in the hilar and mediastinal regions demonstrated high PSMA uptake, with partially increased FDG metabolism.
6. Thickening of the right pleura was observed, with some areas showing high PSMA uptake and slightly elevated FDG metabolism.

During the PET/CT procedures of this case, no adverse effects occurred. Based on the clinical findings from the relevant radiographic and nuclear medicine diagnostic tools, a PSMA PET/CT targeted cognitive fusion puncture biopsy was recommended and performed on October 23, 2023, as described in the following surgical process. Preoperative PSMA PET/CT demonstrated an enlarged prostate with an irregular contour and heterogeneous areas of intense PSMA ligand uptake. The left lobe appeared prominently convex with markedly elevated PSMA uptake. Based on these findings, the left lobe was selected as the primary target for biopsy. During the procedure, the patient was positioned for cystolithotomy. Digital rectal examination revealed a firm prostate with palpable nodules, the mobility between the prostate and the anterior wall of the rectum was acceptable, with no apparent fixation toward bilateral seminal vesicles, and the prostate was obviously fixed. After adequate local surface anesthesia, a transrectal biopsy probe was inserted to visualize both lobes of the prostate. Under cognitive fusion guidance based on PSMA PET/CT imaging, a hypoechoic nodule in the left lobe was identified, and two targeted core needle biopsies were performed. Subsequently, systematic biopsies were obtained from both lobes, yielding a total of ten cores. Biopsy pathology revealed prostatic acinar adenocarcinoma (7/10 cores). The Gleason scores and their respective proportions were as follows: 4 + 5 = 9 (70%), 5 + 4 = 9 (70%), 4 + 5 = 9 (80%), 4 + 4 = 8 (60%), 4 + 5 = 9 (70%), 4 + 4 = 8 (40%), and 4 + 4 = 8 (5%). The immunohistochemical stains were positive for AR and P504S and negative for CK34BE12 and P63. Re-evaluation of the ultrasonography findings in conjunction with MRI revealed that the described sonographic pattern of so-called cauliflower-like bladder mass corresponded to prostatic tumor tissue enlargement with protrusion into the bladder, rather than a primary bladder lesion. Consequently, cystoscopy was deemed unnecessary.

**Figure 4:**
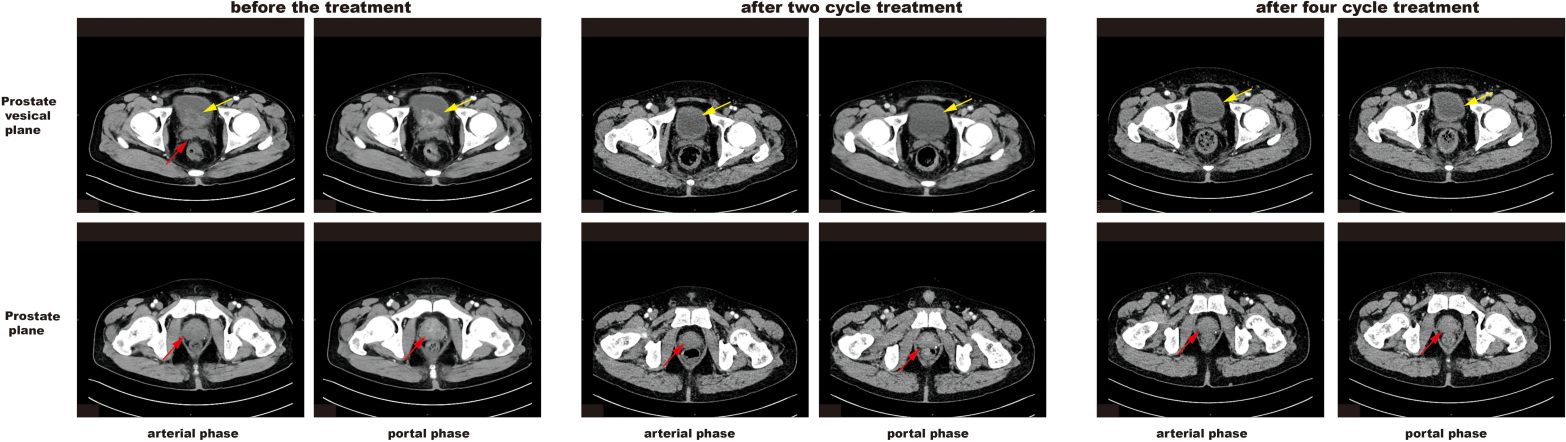
Representative CT images of the primary PCa site before and after treatment. Yellow arrows and red arrows indicate the gradually decreased prostate at the prostate vesical plane and prostate plane, respectively. After four cycles of treatment, the CT images revealed no appreciable abnormal contrast-enhanced lesions.

Radiographic findings suggested bone and lung metastasis from prostate cancer. Because of the multiple metastases in the patient, surgical treatment was not recommended. The patient was treated with six cycles of neoadjuvant chemotherapy (docetaxel 120mg d1 +nedaplatin 65mg d1–2 per 3 weeks). Desumab was further given to inhibit bone destruction. Combined endocrine therapy was subsequently conducted with degarelix and enzalutamide for 2 years. The serum tPSA and fPSA levels decreased to 0.315 ng/mL and 0.048 ng/mL, respectively, after the first cycle of chemotherapy (**Figure 1C**). CT examination revealed that most of the nodules on the lungs and pleura also shrunk (**Figures 3C–E**). The serum tPSA and fPSA maintained a low level during the entire treatment process (**Figure 1C**). Compared with the first chemotherapy session, the osseous lesions did not significantly change throughout the entire treatment period on the CT images. Furthermore, CT images also demonstrated an effective therapeutic response by showing a progressive reduction in prostate size and amelioration of abnormal contrast-enhanced lesions after four treatment cycles (**Figure 4**).

## 3 Discussion

In this case, we report a high-volume, invasive prostate cancer with disproportionately low PSA level, demonstrating the diagnostic and prognostic complexities associated with atypical clinical presentations. Despite a boundary level of tPSA, comprehensive imaging revealed extensive multi-organ involvement, including direct bladder invasion, pulmonary involvement, and multifocal skeletal metastases. Conventional modalities, such as ultrasound, CT and MRI, detect initial structural lesions but fail to fully delineate the metastatic situation or tissue origin. In this case, we creatively applied PSMA PET/CT for cognitive fusion and ultrasound-guided transrectal prostate biopsy. Aiming at the obvious high uptake of the PSMA ligand in the left lobe of the prostate, we targeted the basal part of the left lobe, successfully detected prostate tumor tissue, and laid a pathological foundation for subsequent treatment. Traditional ultrasound-guided transrectal prostate biopsy usually adopts systematic puncture, and the puncture points are evenly distributed on both sides of the prostate. It is difficult for this method to accurately target the suspected lesions on imaging. In this case, with the help of accurate PSMA PET/CT examination, suspicious lesions of the prostate can be efficiently found. Based on such radiographic support, surgeons can initially locate the prostate lesions cognitively, fuse this cognitive localization with the real-time ultrasound imaging during puncture biopsy, identify the lesions accurately and efficiently, and then guide the biopsy needle precisely into the target lesions, thus achieving accurate tumor detection.

Based on the insights from recent advancements in neoplasm realm, it is evident that contemporary oncological practice urgently warrants innovative diagnostic and therapeutic strategies to address complex clinical challenges, such as concealed primary lesion, atypical metastatic patterns, intricate clinical scenarios and recommendations for novel therapeutic approaches (15–17). According to the Chinese Urological Association guidelines, a PSA level below 4 ng/mL is considered normal, whereas values above 10 ng/mL warrant prostate biopsy. For patients with PSA levels between 4 and 10 ng/mL, assessment of the fPSA/tPSA ratio is recommended, and biopsy should be performed when the ratio is below 0.16 (18). However, an f/tPSA ratio < 20–25% is also widely recognized as a high-risk indicator for malignancy in other guidelines. Other studies have shown that approximately 15% of men with PSA levels less than 4 ng/mL may harbor prostate cancer if a biopsy is performed, emphasizing that low PSA does not reliably exclude disease. Moreover, high-grade tumors may produce relatively low PSA despite aggressive pathological features, as demonstrated in this patient, reflecting the nuanced and sometimes paradoxical relationship between PSA levels and oncological biology. Therefore, the patient’s tPSA level (7.25 ng/mL) and fPSA/tPSA ratio (0.19) was sufficient to justify further examinations in this case. Surprisingly, the patient truly showed such a high-volume, invasive prostate cancer with extensive disease burden but disproportionately low PSA level, which adds diagnostic complexity and challenges to contemporary prostate cancer management (19). Collectively, these findings illustrate the limitations of PSA as a solitary diagnostic tool and underscore the necessity for integrated, multimodal diagnostic strategies in contemporary clinical practice (20).

The clinical advantage of PSMA PET/CT in diagnosing PCa was particularly evident in this patient, providing critical insights that conventional radiographic modalities alone could not achieve (21). By targeting PSMA receptors expressed on prostate cancer cells, PSMA PET/CT enables highly sensitive and specific detection of both primary and metastatic lesions (22). In this case, extensive lymph node involvement, multifocal bone lesions, and pulmonary and pleural oncological nodules were identified, all of which demonstrated high PSMA uptake (23). These findings not only confirmed the presence of widespread metastatic disease despite comparatively low PSA level, but also excluded potential differential diagnoses, including primary bladder carcinoma with prostate involvement or the coexistence of synchronous malignancies from different organ origins. Furthermore, PSMA PET/CT revealed metastatic lesions in the sacrum and left pubis with intense PSMA but moderate FDG uptake, which were not clearly identifiable on routine CT images. These findings highlight its diagnostic advantage in revealing early or occult skeletal involvement, which conventional radiographic tools may underestimate. Therefore, the integration of PSMA PET/CT into the diagnostic workflow allows precise disease staging and facilitates tailored therapeutic planning, including endocrine therapy, systemic chemotherapy, and actionable radioligand therapy. This case reinforces the value of PSMA PET/CT as an advanced imaging modality that can overcome the limitations of PSA-based detection and routine radiography, particularly in patients with high-grade, metastatic disease presenting with a disproportionately low PSA. In this case, PSMA PET/CT also showed great value in pathological detection of prostate lesions. PSMA PET/CT can display the prostate lesions with high PSMA uptake more intuitively, so that surgeons can more efficiently fuse the suspicious lesions displayed by PSMA PET/CT with the real-time ultrasound images in ultrasound-guided prostate biopsy, which makes targeted puncture for suspicious lesions in the basal part of the left lobe of prostate more feasible and can effectively improve the detection rate of potential prostate lesions. Especially in patients with slightly elevated PSA, conventional imaging methods show that prostate tumor lesions are insufficient, and the advantages of prostate targeted puncture combined with PSMA PET/CT images are more obvious. In this case, PSMA PET/CT also demonstrated great value in the pathological detection of prostate lesions. The intuitive visualization of prostate lesions through high PSMA uptake enables surgeons to efficiently integrate the suspicious regions identified by PSMA PET/CT with real-time ultrasound images during prostate biopsy, thereby facilitating precise, targeted core needle biopsy of the lesions and effectively improving the detection rate of potential prostate malignancies. The patient tolerated all radiographic diagnostic procedures without adverse events such as allergic reactions, nausea, vomiting, headache, etc. PSMA and FDG PET/CT scans were performed on separate days to minimize potential cumulative effects. Standard post-procedural precautions, including hydration and temporary avoidance of risk close contact, were implemented.

This study is subject to several limitations. First, the diagnostic and therapeutic regimens for this case largely rely on PSMA PET/CT, which, although highly sensitive, may not be universally available in all hospitals or clinical institutions. Second, the patient’s response to treatment was assessed via PSA levels, which may not fully reflect the tumor’s biological behavior, particularly in similar disproportionately low-PSA cases. Last, the retrospective nature of the case also limits the ability to generalize findings, as the whole clinical plan was tailored to this specific patient. Therefore, further prospective studies are needed to confirm the utility of PSMA PET/CT in routine clinical practice for such multiple metastatic PCa patients with atypical PSA profiles.

In conclusion, this case highlights three key points in PCa management. First, a moderate PSA level does not exclude the possibility of high-grade or metastatic prostate cancer. Second, PSMA PET/CT–guided cognitive fusion biopsy integrates high-resolution molecular imaging with real-time transrectal ultrasound, enabling precise localization, targeted sampling and efficient diagnosis of intraprostatic lesions. PSMA PET/CT also provides high sensitivity and specificity for detecting metastases and accurate staging, particularly when accompanied by other high-risk features such as a high Gleason score. Finally, a comprehensive radiographic scheme plays an important role in providing standardized evidence to inform clinical decisions (24). These findings advocate for a multimodal diagnostic strategy that integrates both conventional and advanced diagnostic tools to enhance detection, guide biopsy, improve staging, and optimize personalized treatment in borderline PSA level but high-volume, aggressive prostate cancer patients.

## Data Availability

The raw data supporting the conclusions of this article will be made available by the authors, without undue reservation.
